# A patient with Trisomy 13 mosaicism: review and case report

**DOI:** 10.1186/1753-6561-9-S1-A51

**Published:** 2015-01-14

**Authors:** U Mustaki, S Jackson

**Affiliations:** 1 Royal College of Surgeons Ireland,123 St. Stephen’s Green, Dublin 2, Ireland; 2 Neonatal Intensive Care Unit, National Maternity Hospital, Ireland

## 

Complete Trisomy 13 or Patau’s Syndrome is a relatively common (1/10,000 births) and uniformly fatal chromosomal disorder. In 5% of cases not all cells are trisomic, some cells are euploid [1]. This aberration, known as Trisomy 13 Mosaicism, is not well described but may lead to a milder form of the disease. Descriptive case report and comprehensive literature search of MEDLINE database using PUBMED MeSH terms “mosaicism” “and” “patau syndrome”. A review of references from selected articles was also performed.

A seven-week old girl with an antenatal diagnosis of Trisomy 13 Mosaicism was delivered via an uncomplicated birth to a 35 year old mother of African ethnicity. Dysmorphic features include a third fontanelle, a flat nasal bridge, and polydactyly, clenched fists and “rocker-bottom” feet. An echocardiography revealed mild congenital heart defects. Management was nasal oxygen and nasogastric feeding in NICU. The patient was discharged home at 28 days of life with no medical needs and liaised with the palliative care team. At 6 weeks the patient remained clinically stable, having experienced one clinically suggestive seizure but no significant deterioration in her condition.

This case adds to the currently limited understanding of Trisomy 13 Mosaicism on which we offer an up-to-date review. It discusses the relation of Trisomy 13 mosaicism to the better-known Patau’s Syndrome, particularly with regards to prognosis, and highlights the ethical dilemmas that arise in the management of such patients for whom predicting outcomes has remained extremely challenging to date. In particular, we examine the role of antenatal counseling and the decision of palliation versus active medical management.
